# Population Size Estimation of Men Who Have Sex With Men in Low- and Middle-Income Countries: Google Trends Analysis

**DOI:** 10.2196/58630

**Published:** 2025-01-09

**Authors:** Carly M Malburg, Steve Gutreuter, Horacio Ruiseñor-Escudero, Abu Abdul-Quader, Wolfgang Hladik

**Affiliations:** 1 Division of Global HIV and TB, Centers for Disease Control and Prevention, 1600 Clifton Rd, Atlanta, GA, 30322, United States, 1 8103383534; 2 CDC Global Health Fellowship Program, Public Health Institute, Oakland, CA, United States

**Keywords:** population size estimation, men who have sex with men, MSM, PSE, google trends, HIV, AIDS, programming and policy, internet, porn, gay porn, male adult, geriatric, linear regression, homosexuality, sensitivity analysis, World Health Organization, WHO, epidemiology

## Abstract

**Background:**

Population size estimation (PSE) for key populations is needed to inform HIV programming and policy.

**Objective:**

This study aimed to examine the utility of applying a recently proposed method using Google Trend (GT) internet search data to generate PSE (Google Trends Population Size Estimate [GTPSE]) for men who have sex with men (MSM) in 54 countries in Africa, Asia, the Americas, and Europe.

**Methods:**

We examined GT relative search volumes (representing the relative internet search frequency of specific search terms) for “porn” and, as a comparator term, “gay porn” for the year 2020. We assumed “porn” represents “men” (denominator) while “gay porn” represents a subset of “MSM” (numerator) in each county, resulting in a proportional size estimate for MSM. We multiplied the proportional GTPSE values with the countries’ male adult population (15‐49 years) to obtain absolute size estimates. Separately, we produced subnational MSM PSE limited to countries’ (commercial) capitals. Using linear regression analysis, we examined the effect of countries’ levels of urbanization, internet penetration, criminalization of homosexuality, and stigma on national GTPSE results. We conducted a sensitivity analysis in a subset of countries (n=14) examining the effect of alternative English search terms, different language search terms (Spanish, French, and Swahili), and alternative search years (2019 and 2021).

**Results:**

One country was excluded from our analysis as no GT data could be obtained. Of the remaining 53 countries, all national GTPSE values exceeded the World Health Organization’s recommended minimum PSE threshold of 1% (range 1.2%‐7.5%). For 44 out of 49 (89.8%) of the countries, GTPSE results were higher than Joint United Nations Programme on HIV/AIDS (UNAIDS) Key Population Atlas values but largely consistent with the regional UNAIDS Global AIDS Monitoring results. Substantial heterogeneity across same-region countries was evident in GTPSE although smaller than those based on Key Population Atlas data. Subnational GTPSE values were obtained in 51 out of 53 (96%) countries; all subnational GTPSE values exceeded 1% but often did not match or exceed the corresponding countries’ national estimates. None of the covariates examined had a substantial effect on the GTPSE values (*R*^2^ values 0.01‐0.28). Alternative (English) search terms in 12 out of 14 (85%) countries produced GTPSE>1%. Using non-English language terms often produced markedly lower same-country GTPSE values compared with English with 10 out of 14 (71%) countries showing national GTPSE exceeding 1%. GTPSE used search data from 2019 and 2021, yielding results similar to those of the reference year 2020. Due to a lack of absolute search volume data, credibility intervals could not be computed. The validity of key assumptions, especially who (males and females) searches for porn and gay porn, could not be assessed.

**Conclusions:**

GTPSE for MSM provides a simple, fast, essentially cost-free method. Limitations that impact the certainty of our estimates include a lack of validation of key assumptions and an inability to assign credibility intervals. GTPSE for MSM may provide an additional data source, especially for estimating national-level PSE.

## Introduction

The Joint United Nations Programme on HIV/AIDS (UNAIDS) estimated that in 2022, about 39 million people were living with HIV worldwide [1]. HIV burden is higher among men who have sex with men (MSM), people who inject drugs, sex workers, and transgender persons, which together are often described as key populations (KP) [1]. KPs and their paying or nonpaying sexual partners may account for 70% of new HIV infections worldwide, with an estimated 80% of new HIV infections outside sub-Saharan Africa (SSA) and 55% of all new infections within SSA [1,2].

Key population size estimation (PSE) is needed to estimate the number of individuals belonging to a KP in a given geographical area [3,4]. PSEs provide the denominator values to inform KP programming and policy [5]. However, PSE is a difficult field and its methods often lack rigor in design or implementation, and the many methods available reflect the lack of an acceptable gold standard [3,6]. Challenges to PSE include lack of sampling frames, mobility, and nondisclosure of KP-defining behaviors [3,4]. Further, most PSE methods produce local estimates whereas national PSE estimates are often obtained through “expert opinion,” simple projection, or modeling and less often through national-level empirical data such as direct survey questions or the network scale-up method, both used in general population-based surveys [6,7]. Direct survey questions about KP-defining traits experience reporting bias and require a major effort unless they can be added to an already planned general population survey. The frequent lack of reliable national-level PSE constitutes an even larger challenge compared with the availability of local PSE and complicates national, regional, and global HIV estimation work [3,8-10].

The rise of the internet facilitates web-based activities to improve public health, including in the field of digital epidemiology and infoveillance [11]. Recently, a new PSE method using Google Trends (GT) internet search data was proposed in a proof of concept paper by Card et al [12] GT is a free cloud-based app that displays the relative frequency of user-specified Google search terms as trends across time and user-selected geographical areas [12-14]. Card et al [12] used GT and Canadian census data to estimate the local PSE of MSM in urban and rural locations throughout Canada. Card et al [12] related search terms presumed to be representative of MSM (“gay porn”) to that presumed to be representative of the general (male) population (“porn”). By relating these 2 sets of values, Card et al [12] estimated the relative size of MSM in these Canadian towns. To date, no other published PSE exists using this method.

The literature on pornography consumption by sex and sexual orientation is limited and often the MSM population is not represented. However, a major porn website reported that about a third of its visitors globally in 2021 were reportedly women [15,16]. Further, women, regardless of sexual orientation, may also watch gay porn, possibly in substantial numbers [17]. Beyond this, we found no meaningful gray literature or peer-reviewed articles about internet pornography consumption in low- and middle-income countries (LMICs) or pornography consumption by MSM in LMICs. We are also not aware of (gray) literature about the proportion of heterosexual and homosexual men searching Google for (gay) porn in LMICs.

We expanded the literature search to include high-income settings. A study conducted in the United States reported that more men than women consume pornography (92%:68%, respectively) over the span of a year [18]. The study did not report the type of pornography consumed or disaggregate male respondents by sexual orientation or practice [18]. A separate study from Norway with a sample of some 2300 male and female participants suggested that more men than women consume some pornography (94% of men and 68% of women) [19]. However, only 5% (n=106) of participants identified as gay/lesbian/bisexual, no breakdown of sexual orientation by sex was given, and no information on the type of pornography consumed by participants was available [19].

The aim of this study was to examine the potential utility of using GT data to obtain MSM PSE in selected LMICs.

## Methods

### Preliminary Literature Search

A nonsystematic literature search was conducted to better understand the behavior of pornography consumption of the general population and sexual minorities, by sex, as well as the relative frequencies with which these populations search for (gay) porn in general (via Google) or by directly accessing specific porn sites.

### Selection of Countries

We analyzed GT data for a selected set of 54 countries that receive support from the US President’s Emergency Plan for AIDS Relief, the US Government’s initiative to support global HIV responses, for which information on MSM PSE has been sought [2,20]. These countries are located in SSA (n=29), Asia (n=13), the Americas (n=11), and Ukraine (Tables1  2).

**Table 1.** National men who have sex with men (MSM) population size estimation (PSE) for US President’s Emergency Plan for AIDS Relief supported countries (n=53) using Google Trends (GT) data for the year 2020^a^^,^^b^.

Region and country		GT (number of MSM), n	GT, %	UNAIDS^c^ GAM^d^ regional %, median (IQR)^e^	UNAIDS KP^f^ Atlas, %
East Africa^g^				1.67	
	Burundi	48,500	1.77		0.34
	Ethiopia	365,000	1.28		—^h^
	Kenya	276,000	1.99		0.24
	Rwanda	51,300	1.54		0.15
	Tanzania	243,000	1.73		0.35
	Uganda	154,000	1.47		0.23
Southern Africa^i^				1.67	
	Angola	106,000	1.44		—
	Botswana	13,000	2.12		0.43
	Eswatini	4500	1.57		1.38
	Lesotho	10,000	1.71		1.05
	Malawi	52,500	1.16		0.94
	Mozambique	134,000	1.87		0.22
	Namibia	16,500	2.60		—
	South Africa	393,000	2.46		1.94
	Zambia	51,800	1.18		0.15
	Zimbabwe	53,000	1.64		0.71
West Central Africa^j^				1.28 (IQR 0.45‐1.50)	
	Benin	34,000	1.18		0.20
	Burkina Faso	88,000	1.80		0.07
	Cameroon	148,000	2.29		0.11
	Cote d’Ivoire	166,000	2.68		0.90
	DRC	33,000	1.66		0.98
	Ghana	112,000	1.40		0.69
	Liberia	22,600	1.83		6.04
	Mali	70,500	1.55		0.09
	Nigeria	614,000	1.26		0.49
	Senegal	73,600	1.94		1.38
	Sierra Leone	27,000	1.36		0.16
	Togo	53,100	2.65		0.30
Asia^k^				1.63 (IQR 0.26‐3.10)	
	Burma	664,000	4.53		1.72
	Cambodia	258,000	5.67		1.93
	India	6,460,000	1.18		0.06
	Indonesia	1,180,000	1.61		1.03
	Kazakhstan	137,000	2.99		1.35
	Kyrgyz Rep.	53,000	3.10		0.99
	Lao PDR	53,000	2.73		2.96
	Nepal	83,000	1.19		0.86
	PNG	31,000	1.30		1.58
	Tajikistan	52,000	2.14		0
	Thailand	215,000	1.25		3.08
	Philippines	1,260,000	4.27		2.33
	Viet Nam	1,953,000	7.46		0.98
Europe					
	Ukraine	366,000	3.48	2.11 (IQR 1.75‐2.49)	1.71
Caribbean^l^				2.71	
	Dominican Rep.	124,000	4.26		4.90
	Guyana	8200	3.60		1.45
	Haiti	108,000	3.60		1.03
	Jamaica	24,000	2.91		5.15
	Trinidad and Tobago	11,000	3.04		—
Central and South America^m^				3.37	
	Brazil	2,960,000	5.18		3.50
	El Salvador	85,000	5.20		3.31
	Guatemala	245,000	5.09		2.42
	Honduras	147,000	5.32		1.48
	Nicaragua	114,000	6.32		1.97
	Panama	81,000	7.23		2.65

a These estimates are for descriptive purposes only, to examine issues related to the potential utility of the method proposed by Card et al [12]. They represent the MSM population national population size estimates (percentage of MSM) for the year 2020. The percentage of MSM was calculated by taking the average relative search volume score produced by Google Trends for “gay porn” and dividing it by the average relative search volume score produced by Google Trends for “porn.” MSM population size estimate (number of MSM) was calculated by taking the percentage of MSM population size estimate and dividing it by the total male population (ages 15‐49 years). Key populations (KPs) Atlas percentage of MSM population size estimate was calculated by dividing the absolute MSM population size estimate taken from the United Nations Programme on HIV/AIDS (UNAIDS) KPs Atlas dashboard by the total adult male population (ages 15‐49 years), and then multiplying by 100. The absolute value difference was calculated by subtracting the GT absolute MSM population size estimate value from the KPs Atlas MSM population size estimate absolute value. All absolute values under 10,000 are rounded to the nearest 100. All other absolute values are rounded to the nearest 1000. UNAIDS Global AIDS Monitoring system (GAM) values are regional values transcribed from the UNAIDS open-source Spectrum 6 guide. The countries used to create these regions and respective values may not be in full alignment with the countries included in the population size estimate analysis, therefore direct 1:1 comparisons should not be made. Max:Min ratio: The ratio based on the largest and smallest PSE % value in each region.

b Absolute values are not provided as Google Trends does not provide absolute search frequency values.

c UNAIDS: United Nations Programme on HIV/AIDS.

d GAM: Global AIDS Monitoring system.

e IQR values were included for available regions. Regions without an IQR listed did not have one available.

f KP: key population.

g Max:Min ratio: 1.6 (GT) and 2.3 (UNAIDS KP).

h Not available (data missing for the country).

i Max:Min ratio: 2.2 (GT) and 12.9 (UNAIDS KP).

j Max:Min ratio: 2.3 (GT) and 86.3 (UNAIDS KP).

k Max:Min ratio: 6.3 (GT) and 51.3 (UNAIDS KP).

l Max:Min ratio: 1.5 (GT) and 5 (UNAIDS KP).

m Max:Min ratio: 1.2 (GT) and 2.4 (UNAIDS KP).

**Table 2.** Regional median Google Trends Population Size Estimate, United Nations Programme on HIV/AIDS (UNAIDS) Global AIDS Monitoring system (GAM), and key populations (KP) Atlas for men who have sex with men (MSM) populations for the year 2020.^a^

Region	Median regional percentage MSM population size estimation^b^		
	GT^c^, %	UNAIDS GAM, %	UNAIDS KP Atlas, %
Eastern Africa	1.64	1.67	0.24
Southern Africa	1.68	1.67	0.83
West Central Africa	1.73	1.28	0.40
Asia	2.86	1.63	—^d^
Europe	2.86	2.11	1.47
Caribbean	3.60	2.71	3.17
Central & South America	5.26	3.37	2.54

a Absolute values are not provided as Google Trends does not provide absolute search frequency values.

b Google Trends (GT) and KP Atlas regional estimates only include estimates from included countries with available data (Table 1). UNAIDS GAM data separate regions differently and include countries that vary from our GT or the KP Atlas regional data: UNAIDS GAM includes eastern and southern Africa in 1 estimate and separates Asia and Europe into 2 estimates (1.63% for Asia and the Pacific, 2.11% for Eastern Europe and Central Asia). Region names were not adjusted in the above table to align with GAM data.

c GT: Google Trends.

d Not available.

### Ethical Considerations

No ethics or review board approval or informed consent was obtained or applicable for this work. All data used in this paper are anonymous, aggregate, and publicly available and sourced.

### GT-Based Population Size Estimation

GT provides results based on exact search terms, unlike the “topical” search results that Google’s main search engine provides. GT does not provide absolute search frequency values; instead, GT offers relative search volume (RSV) values across time (eg, 52 wk) in a specified space (eg, Kenya), ie, it normalizes search frequencies for specific search terms (eg, porn) to a range from 0 to 100, where a search term’s maximum frequency (for the specified geographic area and during the specified time frame) is set at 100 and 0 reflects no search for that term [11,13,14]. Importantly, GT allows users to add “comparator” terms (eg, gay porn) next to the main term (eg, porn); the RSV values for such comparator terms are normalized against the main term’s RSV values [13,21]. For the purpose of PSE calculation, the main term “porn” may represent all men whereas the comparator term “gay porn” may be viewed as a subset of men who represent the subpopulation of gay men or MSM. To generate an MSM PSE from the RSV values we divide the comparator RSV value (gay porn) by the larger same-time, same-place RSV value (porn).

### National Size Estimates

PSE data collection was carried out through GT’s application [13]. We applied this analytic approach for the year 2020 using “porn” and “gay porn” as the main and comparator search terms for each of the 54 countries. The time period for data collection was set as the year 2020, the most recent year for which we could obtain all necessary data for this analysis. Weekly RSV values for “porn” and “gay porn” for the year 2020 were exported, summed, and proportional size estimates obtained. For example, for Botswana, the average of the weekly RSV values for “porn” was 78.3, the corresponding average for “gay porn” was 1.66 and the proportional PSE was therefore calculated as 1.66/78.3=2.1%. This was repeated for all countries. We then calculated the absolute Google Trends Population Size Estimate (GTPSE) by multiplying the proportional GTPSE by the total male population aged 15‐49 years in each country, the most used age range for KPs. The sizes for countries’ 15‐49 year-old male general population in 2020 were obtained through Spectrum (version 6.1, Avenir Health).

### Local Size Estimates

GT data can be restricted to subnational areas. Separately from national estimates, for each country, we also attempted to obtain local GTPSE for the political (or, if different, commercial) capital city. Where data were unavailable for the political or commercial capital city, we used data from the district that contained the capital city. The calculation to obtain relative GTPSE was then the same as for the national level. We did not produce absolute subnational GTPSE.

### Consistency of GTPSE Results With WHO-Recommended Minimum Estimate

We assessed whether the GTPSE results met the World Health Organization (WHO) and UNAIDS recommendation that national MSM PSE should represent at least 1% of the general adult male population [22,23].

### Comparability

We compared the country-level GTPSE against 2 reference data sources used by UNAIDS: the KP Atlas database and the Global AIDS Monitoring system (GAM) [22,24,25]. The KP Atlas database stores countries’ self-reported absolute MSM size estimates using a wide range of PSE methods, often projected up to national scale from local estimates, with primary data collected over different periods of time. Proportional KP Atlas PSE values were computed by dividing the absolute MSM PSE values from the KP Atlas over the male general population (15‐49 years). UNAIDS’ GAM is a global data warehousing system that informs policy and facilitates monitoring, including KP size estimates. Using GAM data, UNAIDS curated a table with regional relative MSM PSE (median and IQR) deemed reasonable.

### Covariates Potentially Affecting GTPSE

#### Overview

We examined the potential effect of select covariates on the relative GTPSE values by performing regression analysis for each covariate. The country-specific covariates we examined included internet penetration, urbanization, stigma, and criminalization of homosexuality. The covariate data are provided in Table S1 in Multimedia Appendix 1; these data were not used to adjust GTPSE values.

#### Internet Penetration and Urbanization

Internet penetration data were extracted from the World Development Indicators database through the World Bank and the Internet World Statistics database, indicating the percentage of each country’s total population with access to the internet. Urbanization data were obtained from the World Development Indicators database through the World Bank, indicating the percent of the total population in each country considered urban [26,27].

#### Stigma

Country-level stigma values were extracted from the Global Acceptance Index [28]. This index was developed using computer modeling informed by responses to questions that measure attitudes toward lesbian, gay, bisexual, transgender, or intersex people from 11 different global surveys to create a stigma score in 175 countries toward lesbian, gay, bisexual, transgender, or intersex persons. The system scores countries on a scale of 1 to 10; higher scores indicate less stigma [28].

#### Criminalization

The State-Sponsored Homophobia International Lesbian, Gay, Bisexual, Trans, and Intersex Association report was used to evaluate the effects of criminalization of homosexual orientation or behavior on GTPSE [28]. The report classifies countries based on their level of legal protection or criminalization of sexual orientation and same-sex sexual acts. These classifications, ranging from most severe to most protected, include the death penalty, up to lifelong imprisonment, up to 8 years imprisonment, de facto criminalization, no criminalization or legal protections, limited protections, employment protections, broad protections, and constitutional protections. We converted these classifications into a quantitative ranking ranging from +4 to −4. The most severe classification (death penalty) was assigned the rank value “+4” and descended to the least severe/most protective classification (constitutional protection) with a rank value “−4.”

### Sensitivity Analysis

Using a subset (n=14) of the 53 countries we performed 3 sensitivity analyses at the national level. The 14 countries were randomly selected among countries with prominent languages being French, Spanish, or Swahili. The first sensitivity analysis probed the effect of select non-English search languages. The 14 countries comprised 4 using Swahili (Kenya, Tanzania, Uganda, Democratic Republic of Congo [DRC]), 5 using French (Cote d’Ivoire, Senegal, Cameroon, Mali, Haiti), and 5 using Spanish (Dominican Republic, Panama, El Salvador, Nicaragua, Honduras) as their national/dominant language. We generated GTPSE using search terms in Swahili (“ngono” and “ngono za mashoga”), French (“porno” and “porno gay”), and Spanish (“porno” and “porno gay”) and compared them to the original relative GTPSE values. Using the same 14 countries, the second sensitivity analysis probed the effect of different search terms in English on GTPSE, that is, “sex,” ”gay sex” as well as “sex,” ”anal sex” and compared them to the original GTPSE (porn and gay porn). The third sensitivity analysis probed the effect of using different calendar years, ie, (2019 [pre-COVID] and 2021) and compared them to the original 2020 GTPSE values, using the original English language search terms.

## Results

### GTPSE and Comparability

Of the 54 countries examined, 1 (South Sudan), was omitted for lack of RSV values. All remaining 53 countries had GTPSE exceeding 1% (Table 1), similar to GAM values (all exceeding 1% as well) and compared with KP Atlas values where 24 out of 53 (45%) countries showed values above 1%. GTPSE ranged from 1.16% to 7.46% (median 1.99%, IQR 1.54%‐3.48%), compared with 0.06% to 6.04% (median 0.99%, IQR 0.34‐1.93%) in the KP Atlas, and 1.38% to 2.82% in GAM regions. In 48 out of 53 (91%) countries, relative GTPSE exceeded estimates in the KP Atlas values; KP Atlas values were larger in 5 countries (DRC, Liberia, Lao People’s Democratic Republic [PDR], Thailand, and Jamaica). Absolute differences between GTPSE and KP Atlas ranged from −312,900 (Thailand) to 6,221,800 (India). Table 2 displays regional median GTPSE, ranging from 1.64% (East Africa) to 5.26% (Central/South America), larger in all regions than the corresponding KP Atlas values and largely similar to GAM values in most regions. Table 1 also displays the ratios between the largest and smallest country-level %PSE for each region, separately for GT and KP Atlas values. While substantial variability is seen in all regions and for both data sources (GT and KP Atlas), in all regions the observed heterogeneity was consistently higher for KP Atlas values compared with GT values.

Local GTPSE pertaining to political or commercial capitals or the larger sub-national areas encompassing these are displayed in Table 3. We could obtain local estimates for 51 out of 53 (96%) countries’ capital cities; GT did not provide data for Nairobi (Kenya) and Kathmandu (Nepal). Among the 51 cities with estimates, the GTPSE ranged from 0% to 13% (median 2.2%); most cities’ estimates (44/51, 86%) exceeded 1%. Five cities yielded noncredible GTPSE values of 0%, including Bujumbura (Burundi), Dodoma (encompassing Dar es Salaam, Tanzania), Ouagadougou (Burkina Faso), Monrovia (Liberia), and Vientiane (Laos PDR). Of the 44 subnational GTPSE with values >1%, 18 (41%) were below the same-country national GTPSE.

**Table 3.** Reported local men who have sex with men (MSM) Google Trends Population Size Estimate (GTPSE) (n=53) in the year 2020.^a^

Region and country		Local area^b^	Relative national GTPSE, %	Relative local GTPSE, %	Absolute percentage difference national and local GTPSE, %
East Africa					
	Burundi	Bujumbura	1.77	0	−1.77
	Ethiopia	Addis Ababa	1.28	1.30	0.02
	Kenya	Nairobi	1.99	—^c^	—
	Rwanda	Kigali	1.54	1.70	0.16
	Tanzania	Dodoma	1.73	0	−1.73
	Uganda	Kampala	1.47	1.60	0.13
Southern Africa					
	Angola	Luanda	1.44	2.04	0.60
	Botswana	Gaborone	2.12	0	−2.12
	Eswatini	Mbabane	1.57	1.48	−0.09
	Lesotho	Maseru	1.71	1.01	−0.70
	Malawi	Lilongwe	1.16	2.24	1.08
	Mozambique	Maputo	1.87	2.04	0.17
	Namibia	Windhoek	2.60	2.53	−0.07
	South Africa	Johannesburg (Gauteng)	2.46	0.99	−1.47
	Zambia	Lusaka	1.18	1.56	0.38
	Zimbabwe	Harare	1.64	1.56	−0.08
West Central Africa					
	Benin	Littoral (Cotonou)	1.18	4.11	2.93
	Burkina Faso	Centre (Ouagadougou)	1.80	0	−1.80
	Cameroon	Littoral (Douala)	2.29	2.47	0.18
	Cote d’Ivoire	Abidjan	2.68	1.01	−1.67
	DRC	Kinshasa	1.66	2.04	0.38
	Ghana	Accra	1.40	1.42	0.02
	Liberia	Monrovia	1.83	0	−1.83
	Mali	Bamako	1.55	2.93	1.38
	Nigeria	Abuja (Federal Capital Terriorty)	1.26	1.44	0.18
	Senegal	Dakar	1.94	2.85	0.91
	Sierra Leone	Freetown	1.36	1.01	−0.35
	Togo	Lome	2.65	2.04	−0.61
Asia					
	Burma	Yangon (Yangon Region)	4.53	4.79	0.26
	Cambodia	Phnom Penh	5.67	5.43	−0.24
	India	New Delhi (Uttar Pradesh)	1.18	1.15	−0.03
	Indonesia	Jakarta	1.61	2.20	0.59
	Kazakhstan	Almaty (Almaty Region)	2.99	5.52	2.53
	Kyrgyz Rep.	Bishkek	3.10	3.09	−0.01
	Lao PDR	Vientiane	2.73	0	−2.73
	Nepal	Katmandu/Kantipur	1.19	—	—
	PNG	Port Moresby	1.30	1.01	−0.29
	Tajikistan	Dushanbe	2.14	1.01	−1.13
	Thailand	Bangkok	1.25	3.24	1.99
	Philippines	Manila	4.27	5.51	1.24
	Viet Nam	Hanoi	7.46	4.56	−2.90
Europe					
	Ukraine	Kyiv	3.48	4.14	0.66
Caribbean					
	Dominican Rep.	Santo Domingo	4.26	3.99	−0.27
	Guyana	Georgetown	3.60	3.09	−0.51
	Haiti	Port-au-Prince	3.60	3.09	−0.51
	Jamaica	Kingston (St. Andrew Parish)	2.91	3.38	0.47
	Trinidad and Tobago	Port of Spain	3.04	13	9.96
Central and South America					
	Brazil	São Paulo (State of São Paulo)	5.18	5.92	0.74
	El Salvador	San Salvador	5.20	5.73	0.53
	Guatemala	Guatemala City (Guatemala Department)	5.09	4.91	−0.18
	Honduras	Tegucigalpa (Comayagua)	5.32	8.13	2.81
	Nicaragua	Managua	6.32	5.31	−1.01
	Panama	Panama City	7.23	7.44	0.21

a Absolute values are not provided as Google Trends does not provide absolute search frequency values.

b Local MSM GTPSE for 53 countries for the year 2020 was calculated by restricting the geographic entity to the desired capital city or commercial hub. Where Google Trends (GT) did not provide data for a given city, we substituted the place name with the largest city by population or by district that had data available in GT. This is noted by listing what was available in GT in parenthesis next to the capital city. Kenya and Nepal were excluded from this analysis due to insufficient regional data available in GT.

c Not available (data missing for that country).

### Effect of Covariates

**Figure 1.**
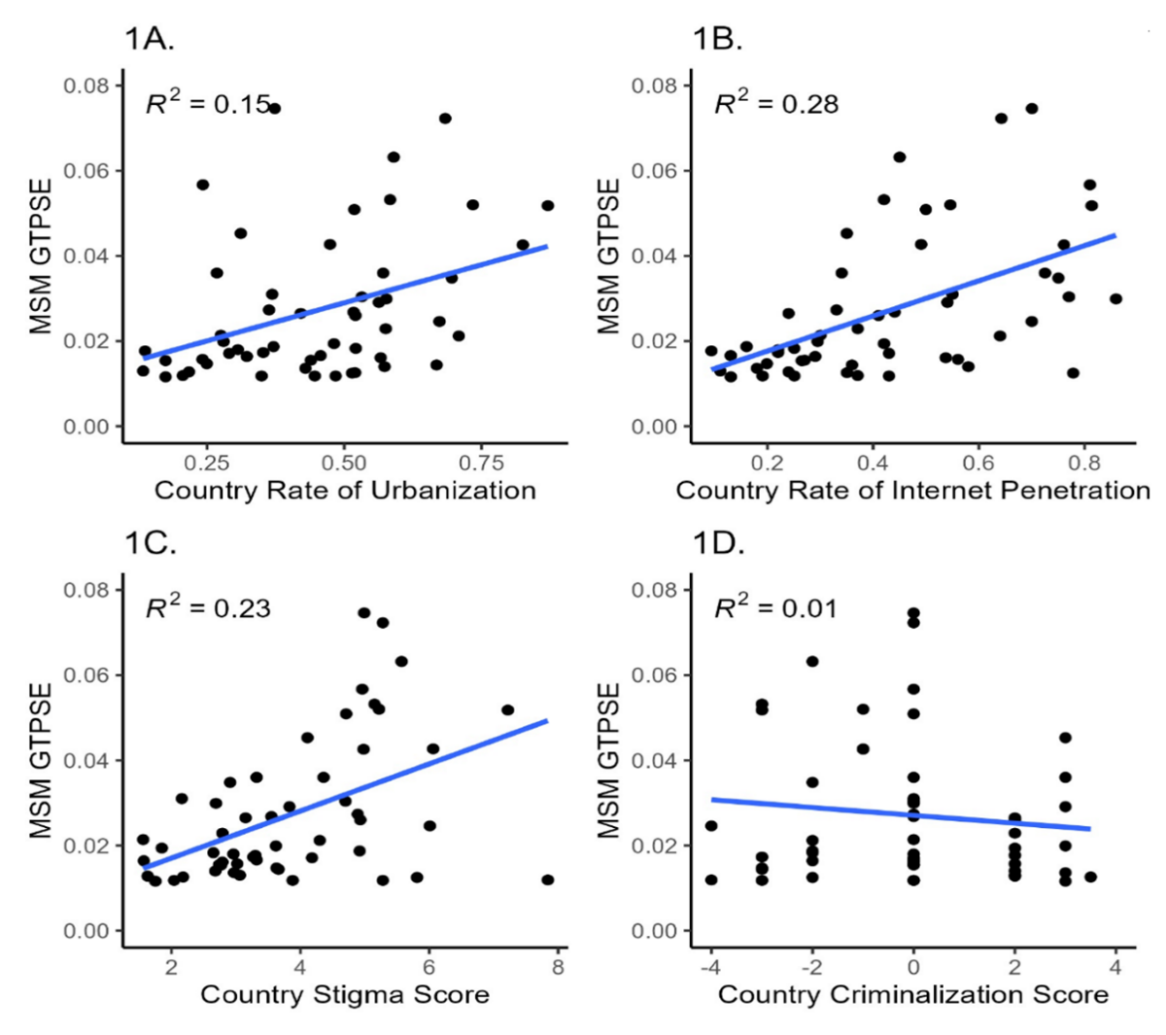
The linear relationship between the Google Trends national population size estimates and the rate of urbanization in each country (n=53). (A) The linear relationship between the Google Trends national population size estimates and the rate of urbanization in each country (n=53). (B) The linear relationship between the Google Trends national population size estimates and the rate of internet penetration in each country (n=53). (C) The linear relationship between the Google Trends national population size estimates and the level of stigma against LGBTQ+ persons in each country (n=53). (D) The linear relationship between the Google Trends national population size estimates and the degree of criminalization against men who have sex with men population in each country (n=53). LGBTQ+: lesbian, gay, bisexual, transgender, queer, and other identities; MSM: men who have sex with men; GTPSE: Google Trends Population Size Estimate.

Figure 1A-D displays the correlations between national-level GTPSE and urbanization, internet penetration, stigma, and criminalization. Coefficients ranged from 0.01 (criminalization) to 0.28 (internet penetration).

### Sensitivity Analysis

Table 4 displays how the GTPSE generated from the alternative search terms compares to the original search term GTPSE. In most countries “Porn/Gay Porn” produced higher PSE values compared with “sex/anal sex” (13/14, 93%) as well as compared with “sex/gay sex” (12/14, 86%). For “sex/gay sex,” all 14 countries produced estimates exceeding 1%. For “sex/anal sex”, 3 out of 14 (21%) countries did not produce estimates reaching the 1% threshold, including Mali for which zero search results were reported for “anal sex.”

**Table 4.** Sensitivity analysis using alternative search terms in Google Trends to calculate national population size estimations (PSEs) for select US President’s Emergency Plan for AIDS Relief countries (n=53) in 2020.^a^

	Original GTPSE^b^	SA alternate search term GTPSE^c^			
	Porn/gay porn PSE	Sex/gay sex PSE	Absolute percentage difference	Sex/anal sex PSE	Absolute percentage difference
Country, %					
Kenya	1.99	1.37	0.62	1.37	0.62
Tanzania	1.73	1.46	0.27	3.54	−1.81
Uganda	1.47	1.38	0.09	1.26	0.21
DRC	1.66	1.55	0.11	1.15	0.51
Cameroon	2.29	1.28	1.01	0.90	1.39
Mali	1.55	1.65	−0.10	0	1.55
Cote d’Ivoire	2.68	1.90	0.78	1.74	0.94
Senegal	1.94	1.50	0.44	0.88	1.06
Haiti	3.60	2.60	1	2.83	0.77
Dominican Rep.	4.26	3.36	0.90	1.83	2.43
Panama	7.23	5.17	2.06	3.71	3.52
El Salvador	5.20	5.34	−0.14	4.19	1.01
Nicaragua	6.32	7.10	−0.78	4.82	1.50
Honduras	5.32	4.85	0.47	2.96	2.36
Median (IQR)	2.49 (1.78-4.97)	1.78 (1.47-4.48)	0.45 (0.10-0.87)	1.79 (1.18-3.40)	1.03 (0.66-1.54)

a Absolute values are not provided as Google Trends does not provide absolute search frequency values.

b GTPSE: Google Trends Population Size Estimate.

c Alternative search terms were chosen based on words that represented the general male population and men who have sex with men subset population in each country (n=53) in the year 2020.

Table 5 shows how GTPSE was generated using alternative language terms compared with the original GT search terms. For Swahili, only 1 country yielded a PSE in that language. All countries using French (n=5), or Spanish (n=5) search terms yielded estimates, all exceeding 1%. All alternative language estimates were lower than the original “porn/gay porn” PSE values.

**Table 5.** Sensitivity analysis using alternate national language searches in Google Trends to calculate national population size estimation for select US President’s Emergency Plan for AIDS Relief countries (n=14) in 2020.^a^

Language and country		Original GTPSE^b^ (English), %	Alternate language term GTPSE, %^c^	Absolute percentage difference, %
Swahili				
	Kenya	1.99	0	1.99
	Tanzania	1.73	0.52	1.21
	Uganda	1.47	0	1.47
	DRC	1.66	0	1.66
French				
	Cameroon	2.29	1.36	0.93
	Mali	1.55	1.07	0.48
	Cote d’Ivoire	2.68	1.35	1.33
	Senegal	1.94	1.28	0.66
	Haiti	3.60	2.23	1.37
Spanish				
	Dominican Rep.	4.26	2.56	1.70
	Panama	7.23	5.14	2.09
	El Salvador	5.20	4.36	0.84
	Nicaragua	6.32	4.13	2.19
	Honduras	5.32	4.07	1.25

a Absolute values are not provided as Google Trends does not provide absolute search frequency values.

b GTPSE: Google Trends Population Size Estimate.

c Alternative language search terms included “ngono/ngono za mashoga” (Swahili), “porno/porno gay” (French), “porno/porno gay” (Spanish).

Table 6 displays how GTPSE generated for alternative years (2019 and 2021) compared with the original 2020 GT searches. All 14 countries in both years produced estimates exceeding 1%. No large discrepancies in PSE between the years were observed; 13 out of 14 in 2019 values were larger than the 2020 values whereas the 2021 values were largely similar to the 2020 values.

**Table 6.** Sensitivity analysis for men who have sex with men population size estimates for select US President’s Emergency Plan for AIDS Relief supported countries (n=14) using Google Trends data in years 2019 and 2021 compared with the year 2020.^a^^b^

	2019 PSE^c^, %	2020 PSE, %	2021 PSE, %
Kenya	2.37	1.99	1.99
Tanzania	1.96	1.73	1.85
Uganda	1.73	1.47	1.69
DRC	1.95	1.66	1.60
Cameroon	2.70	2.29	2.25
Mali	2.30	1.55	2.17
Cote d’Ivoire	2.52	2.68	2.23
Senegal	2.54	1.94	1.90
Haiti	4.33	3.60	2.92
Dominican Republic	4.91	4.26	4.34
Panama	9.36	7.23	6.74
El Salvador	6.19	5.20	4.77
Nicaragua	7.31	6.32	4.93
Honduras	6.79	5.32	5.51

a 2019 and 2021 values were computed in the same way as the reference 2020 estimates.

b Absolute values are not provided as Google Trends does not provide absolute search frequency values.

c PSE: population size estimation.

## Discussion

### Principal Findings

Our analysis suggests that national-level MSM GTPSE is feasible in almost all countries. Importantly, all estimates appeared plausible, that is, they exceeded the WHO/UNAIDS suggested minimum threshold of 1%. Heterogeneity of GTPSE across same-region countries was pronounced within all regions yet smaller than the ratios based on the UNAIDS KP Atlas values which contained numerous PSE values well below the 1% threshold.

Our analysis draws on several strengths. We successfully applied the GTPSE method to many low and middle-income countries, suggesting that GTPSE appears to have wide geographic applicability. We compared the values against 2 PSE data sources at UNAIDS, assessed the potential effect of various covariates on GTPSE values, and conducted a sensitivity analysis with varying English search terms, non-English search languages, and different calendar years. Google is the dominant search engine in all countries covered in this analysis, with a market share ranging between 84% and 99% (data shown in Table S1 in Multimedia Appendix 1) [27]. Although no absolute search volume data were available to us, searches for “porn” globally were among the top 20 search terms in 2023 with about 65 million searches globally each month according to one source [29] although this is still well behind the largest porn site-specific searches. GTPSE may emerge as another example of digital public health and epidemiology that includes real-time surveillance of disease outbreaks [30], assessing the impact of global public health days [31], informing health and health policy research [32], or understanding spatiotemporal patterns of dry eye disease [33].

While most local estimates were plausible (>1%), 14% (n=7) did not reach the WHO/UNAIDS minimum threshold, and 2 more locations did not produce a GTPSE value at all due to lack of GT data and how GT organized the subnational data despite some of the affected cities’ large population sizes. This is not an uncommon finding, as other PSE methods in active use typically do not meet the WHO/UNAIDs minimum threshold. For a few other country or commercial capital cities with no direct GT data available, such as Johannesburg (South Africa), we could obtain a subnational estimate using the larger district or province within which the city (eg, Johannesburg and Pretoria) are located. This may limit the utility and comparability of such local estimates. About one-third of the local (relative) estimates did not reach or exceed the same country national level estimates, somewhat contrary to our expectation that rural-to-urban migration among MSM may be more pronounced than that of other men and so yielding higher GTPSE values [9]. In Card et al’s [12] study on Canadian towns and cities the estimates ranged from 2% to 4% compared with 0% to 13% among the local estimates, whereas the Canadian national estimate was 2.8% compared with 1.2%‐7.5% across all countries we examined. While not a limitation, it is worth noting that weekly RSV data varied widely (data not shown), confirming the recommendation to use GT data for size estimation only over longer time periods, such as a full calendar year.

### Limitations

Like most PSE methods, GTPSE has limitations. In particular, the assumptions underlying the GTPSE method deserve close scrutiny: straight men only search for porn, MSM only search for gay porn, MSM and straight men search for (gay) porn in equal proportions, and women do not search for (gay) porn at all or do not affect the generated GTPSE for MSM. Violations of these assumptions will result in bias if they affect RSV for porn and gay porn to differing extents, hence altering the proportion of porn searches that are directed at gay porn. While the literature from LMIC settings on this topic is very sparse, reports and literature from high-income settings suggest that gay porn is also consumed by heterosexual men and women, suggesting that some bias may be present. Complicating speculations about the magnitude and direction of bias is the fact that specific porn websites’ user statistics may not accurately reflect searches for (gay) porn on Google. Women’s search behavior on Google regarding gay porn may increase or decrease the GTPSE estimates depending on the frequency relative to searches for just porn.

Regrettably, Google does not provide access to its algorithm generating the RSV data nor can users filter GT searches by age or gender. An inherent limitation in using GT data includes the lack of deduplication in the search data (although repeated searches by the same user within a short time period are not counted multiple times by Google) and the lack of absolute search volume data. Not having access to the absolute search volume data impedes the computation of uncertainty intervals (which in most national settings may be expected to be small due to the large search volumes involved). However, absolute search volume information may eventually be made available by Google and is already offered to some extent by select third-party companies. Absolute search volume data may also inform the choice of search language and even search terms and may facilitate composite GTPSE metrics by incorporating multiple GTPSE metrics stemming from different language search terms. Restricting GTPSE-relevant data to male users may further refine GTPSE values by excluding female users, a limitation our analysis could not overcome. VPN (virtual private network) also has the potential to introduce errors if users select a country other than their place of residence. The adoption of VPN may vary considerably across time and by country, and, among US President’s Emergency Plan for AIDS Relief countries. According to one industry website in 2020, VPN was highest in Ukraine (7.9%) and lowest in Kenya (0.5%) [34]. Taken together, these limitations constitute a major source of uncertainty about the bias and precision of GTPSE. For that reason, GPTSE should be regarded as an approximate reference value. Clearly, they do not attain the rigor or transparency of statistically principled estimation from accurately measured data, which the currently best available PSE methods do offer. Additionally, GTPSE may not be feasible for a few countries, perhaps due to poor or little data availability on search terms and frequencies.

GTPSE seems infeasible for size estimation among transgender persons, sex workers, or people who inject drugs. Unlike (gay) porn, where the search is about a web-based product (visual depictions of porn), searches for sex work or clients, transgenderism, or injecting drug use are not directly tied to the internet, and may exhibit a more variable search terminology, and may lack fitting “denominator” search terms (analogous to “porn”).

Overall, the GTPSEs often were substantially higher than the KP Atlas estimates but were more closely aligned with the reported GAM regional estimates. The KP Atlas estimates are based on a broad range of PSE methods typically generating local PSE that may or may not be projected to national scale, or summed or averaged across multiple localities, and may refer to various time points (calendar years) and various age ranges. Many KP Atlas based MSM PSE were implausibly low (<1%), suggesting that substantial differences to GTPSE may often be due to KP Atlas underestimates. The regional GAM estimates are based on a more curated database of PSE after excluding estimates with subpar quality and hence of perhaps more trustworthy quality [22]. However, GAM regions do not exactly overlap with the regions we used for GTPSE and the KP Atlas estimates.

The national MSM GTPSE values were robust against varying levels of urbanization, internet penetration, stigma, and criminalization or protection of homosexuality, negating the need for adjustment and increasing comparability across different settings. The largest influence was seen with internet penetration which can be expected to increase over time. In the sensitivity analysis, the largest differences to the original GTPSE values were seen using alternate English language search terms. Among the 14 examined countries, almost half (43%) of the alternate estimates were below 1% and hence considered implausibly low. This indicates that search term selection is important, especially for comparison across time and space. Further exploration may be warranted to evaluate if country or region-specific English or non-English slang terms may produce plausible estimates; however, the limited sensitivity analysis suggests that “Porn/Gay Porn” may be dependable and consistently produces plausible values. The use of similar search terms in French, Spanish, and Swahili yielded universally lower results; Swahili, not a nationally dominant language in most countries, appears particularly unsuitable as it frequently produced 0% PSE values. As most countries display prominent non-English language use, countries may want to consider using the predominant language (used for web searches) when applying this method while considering any language’s geographic scope in-country. The results also appeared robust across time (two years affected by the COVID pandemic plus 1-year pre-COVID) as the 2 adjacent years produced plausible and (same country) consistent results. The lack of uncertainty intervals however impeded a more meaningful interpretation of the results from the sensitivity analyses.

### Conclusions

Generating national-level PSEs for KPs is challenging for many countries. GTPSE is a simple method with the potential to address this problem efficiently without the need of additional resources. However, the lack of validation of key assumptions and the inability to generate credibility intervals suggest important uncertainty regarding the accuracy and precision of the estimates. Additional research, such as expanding or building on our sensitivity and covariate analysis, to address or better understand these limitations may further improve the quality and utility of GTPSE for MSM in LMICs.

## Supplementary material

10.2196/58630 Multimedia Appendix 1 Supplementary Table 1.

## Abbreviations

GAM: Global AIDS Monitoring system
GT: Google Trend
GTPSE: Google Trend Population Size Estimate
KP: key population
LMIC: low- and middle-income country
MSM: men who have sex with men
PSE: Population size estimation
PSE: population size estimation
RSV: relative search volume
SSA: sub-Saharan Africa
UNAIDS: United Nations Programme on HIV/AIDS
VPN: virtual private network
WHO: World Health Organization
